# Collaboration introduces an innovative practice for incontinence monitoring to provide efficiencies and higher quality of care

**DOI:** 10.1002/alz.090081

**Published:** 2025-01-09

**Authors:** Amy S Chidester

**Affiliations:** ^1^ etectRx, Gainesville, FL USA

## Abstract

Incontinence Management has long been overlooked as a normal part of aging and the national standard of care has become changing individuals every 2 hours. While this was effective for decades, it is no longer providing results and even more challenging for those suffering from Dementia. Over 50% of nursing home residents are incontinent with over 57% of each shift of a certified nursing aide being attributed to incontinence care and management.^1^

Studies show that incontinence is often the root cause of wounds, infections, falls, odor, depression, and behaviors.^2^ Senior care has long provided care in a systematic, uniform way, not allowing for an individualized experience. Instead of making briefs more absorbent, we need to focus on real‐time changes to help mitigate health risks.

Data and science behind predicting when someone will void and helping them not void in an incontinence product is the key. This is the practice of developing and implementing voiding schedules from data that are individualized to each person and providing them with the help they need and deserve. Going further into this concept, the data around patterns of voiding is also extremely powerful for care teams as over time care teams can identify outliers that can indicate to providers a brewing event or condition.

Our practice innovation is a collaboration between an assisted living memory care provider and a digital health sensor platform providing a smart brief that is disposable and remotely “talks” through readers, disguised as art to alert staff to when an individual has had an incontinent episode. Data is collected and reported via a dashboard that provides real‐time alerts for the care team to make decisions on care plans and quality of care can be elevated in ways not available before.

